# Analysis of selected social determinants of health and their relationships with maternal health service coverage and child mortality in Vietnam

**DOI:** 10.3402/gha.v9.28836

**Published:** 2016-02-04

**Authors:** Hoang Van Minh, Kim Bao Giang, Luu Ngoc Hoat, Le Hong Chung, Tran Thi Giang Huong, Nguyen Thi Kim Phuong, Nicole B. Valentine

**Affiliations:** 1 Hanoi School of Public Health, Hanoi, Vietnam; 2 Department of Health Education, Hanoi Medical University, Hanoi, Vietnam; 3 Department of Biostatistics, Hanoi Medical University, Hanoi, Vietnam; 4 Center for Health System Research, Hanoi Medical University, Hanoi, Vietnam; 5 Department of International Cooperation, Ministry of Health, Hanoi, Vietnam; 6 The World Health Organization, Hanoi, Vietnam; 7 Social Determinants of Health (SDH), Public Health, Environmental and Social Determinants of Health Department (PHE), World Health Organization, Geneva, Switzerland

**Keywords:** education, equity, infrastructure, universal health coverage, monitoring, inter-sector

## Abstract

**Introduction:**

Achieving a fair and equitable distribution of health in the population while progressing toward universal health coverage (UHC) is a key focus of health policy in Vietnam. This paper describes health barriers experienced by women (and children by inference) in Vietnam, and measures how UHC, with reference to maternal health services and child mortality rates, is affected by selected social determinants of health (SDH), termed ‘barriers’.

**Methods:**

Our study uses a cross-sectional design with data from the 2011 Vietnam Multiple Indicator Cluster Survey. The study sample includes 11,663 women, aged 15–49 years. Weighted frequency statistics are cross-tabulated with socioeconomic characteristics of the population to describe the extent and distribution of health barriers experienced by disadvantaged women and children in Vietnam. A subset of women who had a live birth in the preceding two years (*n*=1,383) was studied to assess the impact of barriers to UHC and health. Six multiple logistic regressions were run using three dependent variables in the previous two years: 1) antenatal care, 2) skilled birth attendants, and 3) child death in the previous 15 years. Independent predictor variables were: 1) low education (incomplete secondary education), 2) lack of access to one of four basic amenities. In a second set of regressions, a constructed composite barrier index replaced these variables. Odds ratios (ORs) and 95% confidence intervals (95% CI) were used to report regression results.

**Results:**

In Vietnam, about 54% of women aged 15–49 years in 2011, had low education or lacked access to one of four basic amenities. About 38% of poor rural women from ethnic minorities experienced both barriers, compared with less than 1% of rich urban women from the ethnic majority. Incomplete secondary education or lack of one of four basic amenities was a factor significantly associated with lower access to skilled birth attendants (OR=0.28, 95% CI: 0.14–0.55; OR=0.19, 95% CI: 0.05–0.80) and a higher risk of having had a child death in the previous two years (OR=1.71, 95% CI: 1.28–2.30; OR=1.59, 95% CI: 1.20–2.10).

**Conclusions:**

Our study shows the need for accelerating education and infrastructure investments for ethnic minority communities living in rural areas so as to be able to contribute to equity-oriented progress toward UHC.

## Introduction

During the past three decades, economic and social development in Vietnam was accompanied by improvements in maternal and child health. Vietnam is among the group of countries that are on track to achieve several Millennium Development Goals. The poverty rate fell from 58% in 1993 to around 15% in 2008. Infant mortality fell from 44 deaths per 1,000 live births in 1990 to 14 deaths per 1,000 live births in 2011. Under-five mortality decreased from 58 deaths per 1,000 live births in 1990 to 16 deaths per 1,000 live births in 2011. Maternal mortality also decreased from 233 deaths per 100,000 live births in 1990 to 69 deaths per 100,000 live births in 2009, with approximately two-thirds of this decrease related to safer pregnancies (1–4).

Vietnamese health policies show a strong commitment to universal health coverage (UHC) through the development of social health insurance (SHI). The government has devoted significant budgetary resources to expanding population SHI coverage, financed largely through tax subsidies to cover insurance premiums for the poor and other vulnerable groups. The Health Insurance Law passed in 2008, was an important step on the path toward UHC because it integrated the existing health insurance program with the program for the poor, thus bringing together both population groups under a single program. In fact, SHI coverage in Vietnam increased from 30% in 2002 to 68% in 2013, with the SHI benefit package covering most of the outpatient and inpatient services provided in government healthcare facilities (5, 6).

However, despite these successes, progressing toward UHC and improving health equity are still challenges for Vietnam. While two-thirds of the Vietnamese population is covered by health insurance, health insurance spending accounted for only 17% of total health expenditures in 2012 (7). The most challenging issue is to enroll people in the informal sector, including the near poor, and to make SHI an effective mechanism for equitable access to health services and financial protection for all. Out-of-pocket (OOP) payments for healthcare refer to the expenditures made by households when they use health services, primarily for the purchase of drugs, payment of hospital fees, and diagnostic service fees (including self-medication) (8). In Vietnam, over the past 10 years (2000–2010), OOP payments accounted for 50% of total health expenditure, causing catastrophic health expenditure and impoverishment (9). Meanwhile, government subsidies for health are not sufficiently reaching the poor. Hospital subsidies, in particular, have favored the rich, exacerbating existing inequalities (10). Investments in infrastructure and for medical equipment in economically disadvantaged provincial and district health facilities are also inadequate (11). Research in Vietnam shows remaining inequities in maternal and child health outcomes between different segments of the population (12–15). Qualitative studies attribute these inequalities to poverty and lower education as well as to barriers to health service access (the latter of which is traditionally linked to UHC). Access to health services is described as disfavoring the poor, ethnic minorities, and people with low education (12–15).

Internationally, UHC and maternal and child health remain a key area of focus in the third of the sustainable development goals (SDGs) that relate to health. Yet, historical analyses and cross-country literature attribute inequalities in health to factors that create barriers to access health services or living conditions that determine the onset of disease (16–18). Therefore, success in SDG goal 3 will also depend on how well the countries progress on other SDG goals which cover a broad range of health determinants. For example, SDG goal 4 focuses on reducing educational attainment differences between girls and boys.

In view of the SDGs and national health challenges, achieving fairness and equity in many aspects of health, including maternal and child healthcare, will remain a major public policy focus in the Vietnamese health system during the coming years. The 11th National Party Congress Resolution in 2012 highlighted that the “health system in Vietnam should realize social equity in healthcare and that equity must be reflected in specific sectoral mechanisms and policies”. Also in 2012, the Ministry of Health issued the “Master Plan for Universal Health Coverage from 2012–2015 and 2020”. The Master Plan is committed to the goal of expanding SHI coverage along three dimensions: 1) breadth of coverage as defined by enrollment rates; 2) equity and financial protection as defined by the OOP burden on individuals, and 3) the scope of the benefits package (19–22). Yet equity-oriented UHC means not only addressing SHI but also a range of factors that impact on population health. These barriers may arise from other areas of development as reflected in the SDGs (e.g. education, housing) (23).

Therefore, in striving for UHC, it is important for Vietnam “to track factors that enable them to move closer to Universal Health Coverage, both in terms of health systems inputs and outputs, as well as with respect to poverty and other social determinants of health (SDH)” (24). Using data from the Vietnam 2011 Multiple Indicator Cluster Survey (MICS), this paper goes beyond a traditional focus on SHI and poverty, to describe other selected SDH and their relationships with maternal health services and child mortality. These analyses can yield useful insights for tracking equity-oriented progress toward UHC and developing appropriate policy and program responses.

## Methods

### Data source

Data were obtained from the SDH Vietnam 2011 MICS, which was a cross-sectional survey conducted by the General Statistics Office of Vietnam in collaboration with: the Ministry of Health; the Ministry of Labor, Invalids and Social Affairs; the United Nations Children's Fund, and the United Nations Population Fund. The planned sample for the 2011 MICS was 12,000 households in 600 communes/wards of 440 districts in all 63 provinces/cities in the country. This sample was designed to provide reliable estimates of women and children's health at the national level for urban and rural areas, and for the six regions in Vietnam. Of the 12,000 households selected for the sample, 11,642 were present at the time of the survey. Of these, 11,614 successfully completed the interview, resulting in a household response rate of 99.8%. In the interviewed households, 12,115 women (aged 15–49 years) were identified. Of these, 11,663 completed the interview, yielding a response rate of 96.3% compared with eligible respondents in the interviewed households. Women who had a live birth in the preceding two years were asked of questions on antenatal care frequency and access to skilled birth attendants (*n*=1,383). Further information on MICS 2011 can be found elsewhere (25).

## Measurement

### Outcome (dependent) variables

Dependent variables were formulated as dummy variables as follows: 1) antenatal care among women who had a live birth in the preceding two years (yes=1 if a mother had four or more antenatal visits, no=0 otherwise; the cutoff of four was chosen as it is in line with WHO recommendations for coverage completion of antenatal visits, see: www.who.int/gho/maternal_health/reproductive_health/antenatal_care_text/en/); 2) skilled birth attendants among women who had a live birth in the preceding two years (yes=1 if the latest delivery was attended by skilled health personnel, no=0 otherwise), and 3) child deaths (yes=1 if a mother had a deceased child in the previous 15 years, no=0 if all children were surviving). Variables 1) and 2) are commonly aggregated to form ‘service coverage’ indicators, whereas 3) in aggregate form is typically labeled a health outcome or mortality indicator (25).

### Predictor variables

Qualitative research was used to explore the technical feasibility, reliability, validity, and policy and program relevance of different barrier domains (26). We refer to determinants as ‘barriers’ to emphasize the impact they can have on equity in UHC and health. This approach has also been used in the literature (27, 28). We selected two domains specifically focusing on non-financial barriers. These were measured as follows:

1. For the domain education and skills: low maternal education (incomplete secondary education) – if a women aged 15–49 years had not completed secondary education; and
2. For the domain basic infrastructure and amenities: lack of access to one of four basic amenities – if a woman lives in a household, which had no access to either (a) safe energy, (b) safe water, (c) sanitation, or (d) safe energy for cooking.

To investigate options for indicators to track barriers efficiently, we also constructed a composite barrier index which is a combination of the two aforementioned barriers, that is, women aged 15–49 years can be classified as having no barrier, having one barrier, or having both barriers.

These indicators were included as variables in regression analyses. Other variables included as potential confounders in our multiple regression analyses were age, area of residence (urban, rural), ethnicity (Kinh, the majority ethnic group of Vietnam, comprising 86% of the population, and non-Kinh people, who have been previously identified as disadvantaged) (28), and economic condition of the study respondents (16). The economic condition was assessed based on an asset-based wealth index, constructed using principal component analysis. The index was divided into five quintiles as demonstrated elsewhere (29). The confounders were selected because they were available from the MICS data and were expected to be associated with the outcome variables based on an assessment of the Millennium Development Goals (19).

### Data handling and analysis

The MICS 2011 data were cleaned and analyzed using Stata Statistical Software Version 11. Cases with missing data on any independent variable were excluded from the analysis. All analyses were weighted according to the probability of each household unit being sampled to reflect the entire Vietnamese population. Frequency statistics were used to describe the level and distribution of the health barriers in the study population. Multiple logistic regressions were used to assess associations between the selected barriers (each of the two separate barrier variables and the composite barrier index) and variables dependent on maternal healthcare and child mortality.

In total, six regressions were run – three for assessing the relationship between the two selected barrier variables and the maternal service coverage and child mortality outcome variables; and three for assessing the relationship between the composite barrier index and the outcome variables. The model fit when using the composite barrier index was compared with the model fit when using separate barrier variables, based on the log likelihood ratio tests. Odds ratios (ORs) were used to assess the magnitude of associations, and 95% confidence intervals (95% CIs) are reported. Statistical significance was set at *p*<0.05. This study was approved by Ethical Review Board of the Hanoi School of Public Health.

## Results

Table 1 describes the sociodemographic characteristics of the study sample and population: 12.4% were 15–19 years old at the time of the survey; 58.6% had not completed higher secondary education, and 10.4% were ethnic minority people. The proportion of women who lived in rural areas was 67.4%. A higher percentage of women lived in wealthy households than in poor households. Women were not distributed uniformly across households.

**Table 1:** Sociodemographic characteristics of the study sample of mothers aged 15–49 years: frequency, unweighted, and weighted percentages (*n*=11,663)

Characteristics	Frequency	Unweighted percentage	Weighted percentage
Age			
15–19	1,443	12.4	12.4
20–24	1,654	14.2	13.9
25–29	1,638	14.0	14.2
30–34	1,741	14.9	15.5
35–39	1,789	15.3	15.5
40–44	1,629	14.0	13.7
45–49	1,769	15.2	14.7
Education			
None	612	5.3	3.8
Primary	1,883	16.2	16.0
Lower secondary	4,244	36.4	38.8
Upper secondary	2,830	24.3	24.8
Tertiary	2,094	18.0	16.6
Ethnicity			
Ethnic minority	1,827	15.7	10.4
Kinh	9,836	84.3	89.6
Living area			
Rural	6,480	55.6	67.4
Urban	5,183	44.4	32.6
Economic status (asset-based wealth index quintile)			
Poorest	2,152	18.5	16.9
Second	1,924	16.5	18.5
Middle	2,222	19.1	20.8
Fourth	2,529	21.7	21.5
Richest	2,836	24.3	22.3
Total	11,663	100	100

**Fig. 1:**
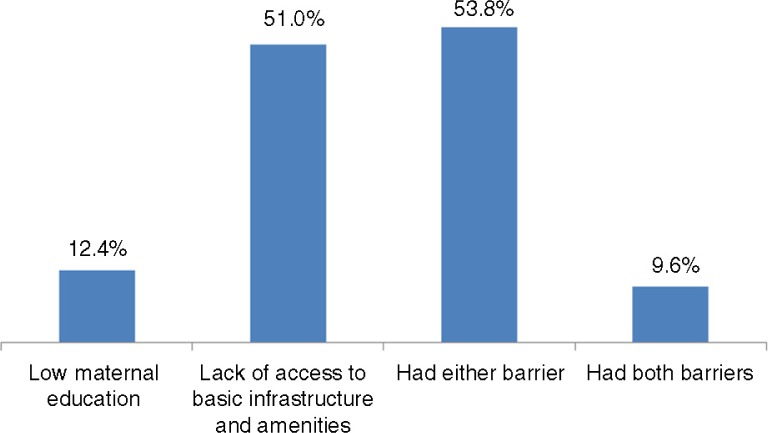
The percentage of Vietnamese women, aged 15–49 years, experiencing one or more barriers to health (*n*=11,663). *Low maternal education*: if a woman aged 15–49 years had not completed secondary education. *Lack of access to basic infrastructure and amenities*: if a woman aged 15–49 years was living in a household which had no access to at least one of the four basic amenities – (a) safe energy, (b) safe water, (c) sanitation, or (d) safe energy for cooking.

Figure 1 shows the barrier indicators. About 12.4% of women had incomplete secondary education; 51% lacked access to one of the four basic amenities; 53% experienced either of these barriers, and 9.6% experienced both barriers. Table 2 shows that these barriers were more prevalent among the disadvantaged women, that is, those living in rural areas, belonging to ethnic minority groups, and having lower economic status. The differences were statistically significant: *p*<0.05; chi-squared test.

**Table 2:** The frequency of health barriers experienced by women aged 15–49 years stratified according to key characteristics (*n*=11,663)

	Low maternal education^a^ (%)	Lack of access to basic infrastructure and amenities^b^ (%)	Had either barrier (%)	Had both barriers (%)
Living area				
Rural	15.0*	64.7*	67.1*	12.6*
Urban	7.1	22.6	26.2	3.6
Ethnicity				
Ethnic minority	33.7*	92.2*	93.4*	32.5*
Kinh	10.0	46.2	49.1	7.0
Economic status (asset-based wealth index quintile)				
Poorest	29.9*	99.7*	99.7*	29.9*
Second	16.0	91.7	92.6	14.9
Middle	11.5	59.9	63.8	7.6
Fourth	7.2	17.6	23.8	1.1
Richest	2.2	4.4	6.5	0.1
Total	12.4	51.0	53.8	9.6

* Statistical significance: *p*<0.05, chi-squared test.

a Low maternal education: if a woman aged 15–49 years had not completed secondary education;

b lack of access to basic infrastructure and amenities: if a woman aged 15–49 years is living in a household which has no access to at least one of the four basic amenities – (a) safe energy, (b) safe water, (c) sanitation, or (d) safe energy for cooking.

Table 3 shows barrier indicators for two constructed population sub-groups with intersecting disadvantage. In the disadvantaged sub-group, i.e. those who lived in rural areas belonged to an ethnic minority and had the lowest economic status: 38.2% experienced educational barriers, 99.6% experienced infrastructural barriers, 99.6% experienced either barrier, and 38.2% experienced both barriers. This contrasts with the advantaged sub-group. The differences were statistically significant: *p*<0.05, chi-squared test.

**Table 3:** Distribution of the health barriers cross-tabulated with two population sub-groups defined by intersecting aspects of socioeconomic status (*n*=11,663)

	Health barrier			
	
Sub-group defined by intersecting aspects of socioeconomic status	Low maternal education (incomplete secondary education)^a^ (%)	Lack of access to basic infrastructure and amenities^b^ (%)	Had either barrier (%)	Had both barriers (%)
Rural, ethnicity minority and poorest	38.2*	99.6*	99.6*	38.2*
Urban, Kinh and richest	2.0	4.4	6.3	0.1
Total	12.4	51.0	53.8	9.6
Gap in absolute percentage points	36.2	94.8	93.3	38.1

* Statistical significance: *p*<0.05, chi-squared test.

a Low maternal education: if a woman aged 15–49 years had not completed secondary education

b lack of access to basic infrastructure and amenities: if a woman aged 15–49 years is living in a household which has no access to at least one of the four basic amenities – (a) safe energy, (b) safe water, (c) sanitation, or (d) safe energy for cooking.

Table 4 reports ORs and 95% CIs from the multiple logistic regression analyses. After adjusting for other confounding factors (i.e. age, living area, ethnicity, and economic status of the women) and holding all other independent variables constant, incompletion of secondary education by mothers was significantly associated with a lower odds of having skilled birth attendants (OR=0.28, 95% CI: 0.14–0.55) and a higher odds of having a child death (OR=1.71, 95% CI: 1.28–2.30). Lacking access to one of four basic amenities was significantly associated with a lower odds of having four or more antenatal visits (OR=0.57, 95% CI: 0.39–0.82), to lower odds of having skilled birth attendants (OR=0.19, 95% CI: 0.05–0.80), and a higher odds of having a child death (OR=1.59, 95% CI: 1.20–2.10). Access to skilled birth attendants was more sensitive to barriers than was antenatal care (i.e. non-significant result found for education).

**Table 4:** Odds ratios for three multiple logistic regressions of low maternal education and lack of access to basic infrastructure and amenities on antenatal healthcare, skilled birth attendants, and child death

	Dependent variables^a^					
	
	Antenatal care (among women who had a live birth in the preceding 2 years) (*n*=1,265)		Skilled birth attendants (among women who had a live birth in the preceding 2 years) (*n*=1,362)		Child death (women who had a deceased child in the previous 15 years) (*n*=11,657)	
			
Independent variables^b^	Odds ratio^c^	95% CI	Odds ratio^c^	95% CI	Odds ratio^c^	95% CI
Low maternal education (incomplete secondary education)						
Yes	0.66	0.43–1.01	0.28*	0.14–0.55	1.71*	1.28–2.3
No	1	1	1	1	1	1
Lack of access to basic infrastructure and amenities						
Yes	0.57*	0.39–0.82	0.19*	0.05–0.8	1.59*	1.2–2.1
No	1	1	1	1	1	1

* Statistical significance: *p*<0.05 level, chi-square test.

a Dependent variables included: 1) antenatal care (yes=1 if a mother aged 15–49 years, who gave birth in the previous 2 years, had four or more antenatal visits, no=0 otherwise); 2) skilled birth attendants (yes=1 if the latest delivery was attended by a health professional, no=0 otherwise); and 3) child death (yes=1 if a mother had a child dead, no=0 otherwise)

b independent variables included: 1) low maternal education (yes=1 if a mother aged 15–49 years, who gave birth in the previous 2 years, or who had had a child who died in the previous 15 years, had not completed secondary education; 2) lack of access to basic infrastructure and amenities if a household has no access to at least one of the four basic amenities (a) safe energy, (b) safe water, (c) sanitation, (d) safe energy for cooking; and other potential confounders (age, living area, ethnicity, and economic status of the women)

c odds ratios are adjusted for age, living area, ethnicity, and economic status of the women.

Table 5 reports ORs and 95% CIs for the composite barrier index. Having both barriers (low maternal education and lack of access to basic amenities) was statistically associated with a lower odds of having four or more antenatal visits (OR=0.39, 95% CI: 0.23–0.66), a lower odds of being attended by skilled health staff (OR=0.06, 95% CI: 0.01–0.29), and a higher odds of having a child death (OR=2.71, 95% CI: 1.87–3.92).

**Table 5:** Odds ratios for three multiple logistic regressions of the composite barrier index on maternal healthcare, skilled birth attendants, and child death

	Dependent variables^a^					
	
	Antenatal care (among women who had a live birth in the preceding 2 years) (*n*=1,265)		Skilled birth attendants (among women who had a live birth in the preceding 2 years) (*n*=1,362)		Child death (women who had a deceased child in the previous 15 years) (*n*=11,657)	
			
Independent variables^b^	Odds ratio^c^	95% CI	Odds ratio^c^	95% CI	Odds ratio^c^	95% CI
Composite index^c^						
Had both barriers	0.39*	0.23–0.66	0.06*	0.01–0.29	2.71*	1.87–3.92
Had either barrier	0.58*	0.39–0.87	0.56	0.11–2.78	1.6*	1.19–2.18
No barrier	1	1	1	1	1	1
*p* values for log likelihood ratio tests comparing the model using the composite barrier index and the one using the two separate barriers	*p*>0.05		*p*>0.05		*p*>0.05	

* Statistical significance: *p*<0.05 level, chi-square test.

a Dependent variables included: 1) antenatal care (yes=1 if a mother had four or more antenatal visits, no=0 otherwise); 2) skilled birth attendants (yes=1 if the latest delivery was attended by a health professional, no=0 otherwise); and 3) child death (yes=1 if a mother had a child dead, no=0 otherwise);

b independent variables included: 1) composite index (both barriers=1 if women had incomplete secondary education and lack of access to at least one of the four basic amenities, had either barrier=2 if women had either barrier, or no barrier=0; the composite index was included as substitutes for the two separate barrier variables); and other potential confounders (age, living area, ethnicity, and economic status of the women)

c odds ratios are adjusted for age, living area, ethnicity, and economic status of the women.

## Discussion

This paper complements existing literature describing maternal and child health in Vietnam which has a strong focus on poverty and health insurance, by assessing other intersectoral barriers to health and UHC. To the best of our knowledge, it provides a first peer-reviewed quantitative analysis of non-financial factors affecting both maternal healthcare services and child health outcomes in Vietnam in 2011.

Intersectoral indicators of barriers need to have relevance for health services and health outcomes and for other sectors. From an equity perspective, these indicators also need to demonstrate relevance for more disadvantaged population groups. From the empirical analyses presented in this paper, the barrier indicators chosen fulfilled both criteria in the context of women and children's health in Vietnam. We identified a sub-group of women, who were likely to experience more disadvantages with respect to health, based on literature discussed previously. These women were from ethnic minorities and poorer households in rural areas. Our study confirmed that they did experience much higher rates of educational and infrastructural barriers than those in the comparison group (Kinh majority, urban, and wealthier households) (23, 28). Furthermore, experiencing barriers was strongly associated with both lower utilization of maternal healthcare services and poorer child health outcomes.

The paper tested a composite barrier indicator, which combined measures of each barrier into a single index. The advantage of using a single number in the context of monitoring is that it enables streamlining of briefings to policy makers. The model fit for the regressions was reassuring.

Historical analyses of ‘good health at low cost’ from Sri Lanka, for example, and also from Cuba make the case for deriving health benefits from an intersectoral approach (30). The historical analyses and results of our research imply that, in moving forward in Vietnam, programs and health services addressing women and children's health could benefit from having more of a multi-sectoral character beyond child survival campaigns and immunization. It may, therefore, not be unreasonable for the health sector to consider intersectoral actions related to championing rapid investment in education, water, sanitation, and energy in rural areas as part of UHC in Vietnam. This approach would also complement the focus on SHI described in the introduction to the paper.

Our findings are important because they add to the existing Vietnamese and international literature on the association between health and factors determining health as well as their equity implications. Lower education is associated with poorer knowledge on health matters and barriers to communication (28), leading to increases in risky health behaviors. This barrier domain also affects the effective availability and accessibility of health services: health services can become available and accessible only if people know of their existence and have the power to access them (30). The education of women in particular is a strong predictor of the health of children and their access to health services (30–36). Other studies also show the importance of the general availability of sanitation to households for explaining access to maternal healthcare services as well as child mortality (37–39).

It is somewhat reassuring that other studies in Vietnam showed similar health mortality inequities as did this study. Elsewhere, child mortality rates were also higher for women residing in rural areas, belonging to ethnic minorities, and with lower economic status (12, 13, 40). Our findings on the incidence of education and barriers to amenities were also in line with previous studies in Vietnam, which showed that inequalities in women's education and living conditions were worse for women residing in rural areas, belonging to ethnic minorities, and with lower economic status (12, 13).

Our paper showed the impacts of barriers on child mortality and access to antenatal care services. However, based on available literature (32), we think it would be reasonable to assume that the barriers we describe could also impact on other health services and health outcomes. For example, a recent meta-study of access to basic amenities also reported high association with *maternal* mortality (39). Further assessments in Vietnam are needed.

A surprising finding was that skilled birth attendance coverage was more strongly associated with women lacking at least one basic amenity than with incomplete secondary education. The role of basic amenities with regard to this specific health service, needs further exploration. In the health facility, water and energy, alongside skilled birth attendants, are highly relevant to user-perceived and actual quality of services. However, the basic amenities variable measured household and not health facility access to amenities. There may be some correlation between household and health facility access to amenities which, in part, explains our results. But beyond this, how does amenities access in the household influence a woman's access to skilled birth attendants?

The issue of combined, or intersecting, incidence of barriers in particular population groups cannot be ignored. There were markedly higher odds (almost double) of women having at least one child death if they experienced both barriers. Health services need to make specific efforts to tailor programs, perhaps working with community surveillance networks, to address these intersectoral causes of mortality. A strong argument could be made for having overall district budgets that are sensitive to changes in the health barrier indicators to prevent child deaths.

In the context of the sustainable development agenda, reaching the health goal “Ensure healthy lives and promote well-being for all at all ages” will require intersectoral action and, therefore, intersectoral monitoring. Indicators that demonstrate a significant contribution of other sectors and other SDGs are noted in this paper. The SDGs on education (SDG 4, “Ensure inclusive and equitable quality education and promote lifelong learning opportunities for all”) and gender equality (SDG 5, “Achieve gender equality and empower all women and girls”) promote targets related to access to quality education and non-discrimination of girls. Clearly, our results confirm the importance of an indicator on incomplete secondary education, which is relevant to education and to the health goal. On the contrary, our basic amenities indicator combines aspects of SDG 6 (“Ensure availability and sustainable management of water and sanitation for all”), SDG 7 (“Ensure access to affordable, reliable, sustainable, and modern energy for all”), and SDG 11 (“Make cities and human settlements inclusive, safe, resilient and sustainable”) and has also been shown to be highly relevant to both UHC and child mortality. Data for the indicators used in this paper should be freely available for other countries, given the wide implementation of MICS and DHS surveys including questions that can be used to develop similar barrier indicators.

We need to note some limitations to the interpretation of our findings based on our chosen methodology. We selected only two barrier indicators for analysis in this paper. We felt that these two indicators would be understandable for both policy makers and community actors and would also complement existing UHC work, as mentioned earlier. Also, both indicators were shown to be feasible, reliable, valid, and relevant to policy/program in tracking progress toward UHC in Vietnam (41). This being said, future work would need to look at additional barrier indicators, which could also imply the use of community-level and specific policy-level variables, as well as other related methodological designs, which could include multi-level analysis. The same observations are also relevant to health and health coverage indicators. A broader set could be explored. While observing these limitations, we note that addressing them implies a far larger data gathering endeavor than the one undertaken for this paper. We also note that the study's cross-sectional design limits interpretation with regard to the importance of determinants over time in the Vietnamese context.

## Conclusions

Barriers to the health of women and children in Vietnam were incomplete secondary education and lack of access to one of four basic amenities. These factors are, as elsewhere, influenced by policies beyond the health sector but they profoundly affect the effectiveness of health services and impact on health equity. The health sector needs to track these factors and associated policies to promote action on the SDH. Without action on health determinants, equity-oriented progress toward UHC will be impeded.

## References

[1] Ministry of Health of Vietnam, Health Partnership group (2011). Joint annual health review 2011.

[2] Ministry of Health of Vietnam (2010). Plan for the protection, care and promotion of the people's health 2011–2015.

[3] UN Inter-agency group for child mortality estimation (2011). Levels and trends in child mortality.

[4] WHO, UNICEF, UNFPA and The World Bank (2012). Trends in maternal mortality: 1990 to 2010. WHO, UNICEF, UNFPA and The World Bank estimates 2012.

[5] Government of Vietnam (2005). Decree 63: issuing health insurance regulation.

[6] Ministry of Health of Vietnam, Health Partnership group (2010). Join annual health review 2010: Vietnam's health system on the threshold of the five-year plan 2011–2015.

[7] Ministry of Health of Vietnam (2011). National health accounts in Vietnam 2009.

[8] Ministry of Health of Vietnam, Health Partnership group (2008). Joint annual health review 2008: health financing in Vietnam.

[9] Levin CE, Van Minh H, Odaga J, Rout SS, Ngoc DN, Menezes L (2013). Delivery cost of human papillomavirus vaccination of young adolescent girls in Peru, Uganda and Vietnam. Bull World Health Organ.

[10] Somanathan A, Dao HL, Tien TV (2013). UNICO studies series 24: integrating the poor into universal health coverage in Vietnam.

[11] Ministry of Health, Health Partnership Group (2013). Joint annual health review 2013.

[12] Viet Nam and the MDGs http://www.undp.org.vn/mdgs/viet-nam-and-the-mdgs/.

[13] Goland E, Hoa DT, Malqvist M (2012). Inequity in maternal health care utilization in Vietnam. Int J Equity Health.

[14] World Health Organization (2005). Maternal mortality in Vietnam 2000–2001: an in-depth analysis of causes and determinants: maternal mortality in Vietnam 2000–2001: an in-depth analysis of causes and determinants.

[15] Knowles JC, Bales S, Cuong LQ, Oanh TTM, Luon DH (2009). Health equity in Vietnam: a situation analysis focused on maternal and child mortality.

[16] Peters DH, Garg A, Bloom G, Walker DG, Brieger WR, Hafizur Rahman M (2008). Poverty and access to health care in developing countries. Ann N Y Acad Sci.

[17] Colgrove J (2002). The McKeown thesis: a historical controversy and its enduring influence. Am J Public Health.

[18] Schuftan C (1990). The child survival revolution: a critique. Fam Pract.

[19] Ministry of Health (2012). Health-related millennium development goals Vietnam 2012.

[20] Central Party of Vietnam (2014). Resolution no 33- NQ/TW dated 9/06/2014 at the 9th conference of the Central party committee No XI referring to ‘Development of culture, human resources to respond to the need of sustainable development of the country’.

[21] National Assembly N-Q (2004). Law of Vietnam on protection, care and education of children dated 15th June 2004.

[22] National Assembly N-Q (2008). Law of Vietnam on health insurance, dated 14th November 2008.

[23] Palmer M, Mitra S, Mont D, Groce N (2014). The impact of health insurance for children under age 6 in Vietnam: a regression discontinuity approach. Soc Sci Med.

[24] World Health Organization, World Bank (2014). Monitoring progress towards universal health coverage: framework, measures and targets.

[25] General Statistics Office (2011). Vietnam multiple indicator cluster survey.

[26] Hoang Van M, Kim Bao G, Nguyen Thi KP, Le Hong C Poster on “Monitoring intersectoral factors influencing equity-oriented progress towards universal health coverage (UHC) and health equity: Vietnam case study.”.

[27] Kushel MB, Gupta R, Gee L, Haas JS (2006). Housing instability and food insecurity as barriers to health care among low-income Americans. J Gen Intern Med.

[28] Malqvist M, Hoa DT, Liem NT, Thorson A (2013). Ethnic minority health in Vietnam: a review exposing horizontal inequity. Glob Health Action.

[29] Rahman M, Haque SE, Mostofa M, Tarivonda L, Shuaib M (2011). Wealth inequality and utilization of reproductive health services in the Republic of Vanuatu: insights from the multiple indicator cluster survey, 2007. Int J Equity Health.

[30] Irwin A, Scali E (2010). Action on the social determinants of health: learning from previous experiences. Social determinants of health discussion paper 1 (Debates).

[31] World Health Organization (2013). A framework for monitoring the social determinants of health equity in relation to universal health coverage. Drawing from the fields of social epidemiology, gender equality and human rights (Draft report).

[32] O'Connell TS, Bedford KJ, Thiede M, McIntyre D (2015). Synthesizing qualitative and quantitative evidence on non-financial access barriers: implications for assessment at the district level. Int J Equity Health.

[33] Rajna PN, Mishra AK, Krishnamoorthy S (1998). Impact of maternal education and health services on child mortality in Uttar Pradesh, India. Asia Pac Popul J.

[34] Alexander GR, Chadwick C, Slay M, Petersen DJ, Pass M (2002). Maternal and child health graduate and continuing education needs: a national assessment. Matern Child Hlth J.

[35] Boyd NR, Windsor RA (2003). A formative evaluation in maternal and child health practice: the Partners for Life Nutrition Education Program for pregnant women. Matern Child Health J.

[36] Boyle MH, Racine Y, Georgiades K, Snelling D, Hong S, Omariba W (2006). The influence of economic development level, household wealth and maternal education on child health in the developing world. Soc Sci Med (1982).

[37] Gurung G (2010). Investing in mother's education for better maternal and child health outcomes. Rural Rem Health.

[38] Benova L, Cumming O, Campbell OM (2014). Systematic review and meta-analysis: association between water and sanitation environment and maternal mortality. Trop Med Int Health.

[39] Fink G, Gunther I, Hill K (2011). The effect of water and sanitation on child health: evidence from the demographic and health surveys 1986–2007. Int J Epidemiol.

[40] Malqvist M, Hoa DT, Thomsen S (2012). Causes and determinants of inequity in maternal and child health in Vietnam. BMC Public Health.

[41] Blas E, Ataguba JE, Huda TM, Bao GK, Rasella D, Gereck MR (2016). The feasibility of measuring and monitoring social determinants of health and the relevance for policy and programme – a qualitative assessment of four countries. Glob Health Action.

